# Low-Cost Fiber Chopped Strand Mat Composites for Compressive Stress and Strain Enhancement of Concrete Made with Brick Waste Aggregates

**DOI:** 10.3390/polym14214714

**Published:** 2022-11-03

**Authors:** Panuwat Joyklad, Panumas Saingam, Nazam Ali, Ali Ejaz, Qudeer Hussain, Kaffayatullah Khan, Krisada Chaiyasarn

**Affiliations:** 1 Department of Civil and Environmental Engineering, Faculty of Engineering, Srinakharinwirot University, Nakhonnayok 26120, Thailand; 2 Department of Civil Engineering, School of Engineering, King Mongkut’s Institute of Technology Ladkrabang, Bangkok 10520, Thailand; 3 Department of Civil Engineering, University of Management and Technology, Lahore 54000, Pakistan; 4 Center of Excellence in Earthquake Engineering and Vibration, Department of Civil Engineering, Chulalongkorn University, Bangkok 10330, Thailand; 5 Department of Civil and Environmental Engineering, College of Engineering, King Faisal University, Al-Hofuf P.O. Box 380, Saudi Arabia; 6 Thammasat Research Unit in Infrastructure Inspection and Monitoring, Repair and Strengthening (IIMRS), Thammasat School of Engineering, Faculty of Engineering, Thammasat University Rangsit, Klong Luang, Pathumthani 12121, Thailand

**Keywords:** recycled brick aggregate, glass fiber chopped sheets, peak compressive stress, ultimate strain, analytical models, regression

## Abstract

Given the excessive demolition of structures each year, the issues related to the generated structural waste are striking. Bricks being a major constituent in the construction industry, also hold a significant proportion of the construction waste generated annually. The reuse of this brick waste in new constructions is an optimal solution considering cost-effectiveness and sustainability. However, the problems related to the substandard peak stress and ultimate strain of concrete constructed with recycled brick aggregates (CRAs) limit its use in non-structural applications. The present study intends to improve the unsatisfactory mechanical characteristics of CRAs by utilizing low-cost glass fiber chopped strand mat (FCSM) sheets. The efficacy of FCSM sheets was assessed by wrapping them around CRA specimens constructed with different concrete strengths. A remarkable increase in the peak compressive stress and the ultimate strain of the CRA specimens were observed. For low, medium, and high strength CRAs, the ultimate strain improved by up to 320%, 308%, and 294%, respectively, as compared to the respective control specimens. Several existing analytical models were utilized to predict the peak compressive stress and ultimate strain of the CRAs strengthened using FCSM sheets. None of the considered models reproduced experimental results accurately. Therefore, equations were formulated using regression predicting the peak stress and ultimate strain of the CRAs confined with FCSM sheets. The predicted values were found to correlate well with the experimental values.

## 1. Introduction

The rapid urbanization and the consequent demolition of existing buildings have raised some serious concerns regarding the proper and safe disposal of construction waste. The risk of an increased carbon footprint looms if proper and adequate measures are not taken regarding the generated construction waste. One possible solution to prevent the costs related to the disposal of construction waste is to reuse it. This not only increases the economic feasibility of the project but also lowers the demand for the rapidly depleting natural aggregate resources [1]. Several studies have highlighted the potential of concrete constructed with recycled aggregates (CRAs) with the aim of its salient features, including its low-cost, sustainability, and environmentally-green solution [2,3,4,5,6,7].

Clay bricks constitute a major role in the construction industry, ascribed to their inexpensive nature and easy availability. As a result, the construction waste generated each year involves a significant portion of clay bricks. For instance, it has been reported that the clay brick waste generated annually in China is increasing in geometric progression [8] and approximately 15 million tons of concrete and brick waste is generated each year in China [9]. Further, roughly 1 billion tons of waste, mainly comprising bricks, is annually produced in the European Union [10]. Therefore, there exists a natural urge to reuse this waste in construction applications to avoid issues related to their disposal. A general consensus is that optimal advantages are associated with the use of recycled aggregates as compared to the use of natural aggregates in concrete [11,12,13,14]. Ohemeng et al. [2] concluded that the production of 1 ton of recycled aggregate concrete was 40% cheaper than the cost of natural aggregate concrete having the same volume. In addition, recycled aggregate concrete resulted in a 97% better environmental impact as compared to that of natural aggregate concrete [2]. However, the mechanical properties of CRA must be determined beforehand to assess its feasibility in structural and non-structural applications. At present, CRA finds its applications mainly in road bases and back fillings ascribed to its substandard mechanical properties that prevent its use in new structural concrete applications [15,16]. The main catalyst for these substandard mechanical properties of CRA has been identified in the mortar that is adhered to recycled aggregates [17]. Concrete made with recycled aggregates tends to absorb more water compared with that of concrete constructed with natural aggregates (CNAs), ascribed to the porous nature of the mortar attached to the recycled aggregates [18]. However, a minimal drop in the compressive strength of CRA is reported for the case of the replacement ratio of natural aggregates below 30% [19,20,21].

External confinement is a technique in which additional materials are applied or wrapped around the concrete members to alter the structural performance of the concrete members. It has been known widely that the external confinement on CNA improves its mechanical characteristics, mainly the peak compressive strength and the ultimate strain [22,23,24,25,26]. Recently, synthetic fiber-reinforced polymer (FRP) jackets gained importance attributed to their excellent confinement characteristics [27,28,29,30]. Synthetic FRP jackets have also been reported to enhance the mechanical properties of CRAs [31,32,33]. Two concerns are identified with the use of synthetic FRP jackets: (1) the production of synthetic FRP jackets includes chemicals that can affect the skin [34,35], and (2) these jackets are expensive and may not justify their costs for low-budget strengthening projects [36,37,38]. This has urged researchers to move towards low-cost and sustainable replacements of the synthetic FRPs. Glass fiber chopped strand mat (FCSM) sheets may be a low-cost and environmentally friendly alternative to synthetic FRP jackets. FCSM sheets are recognized for their easy availability and durability [39,40]. Jeffrey et al. [40] investigated the residual strength of FCSMs due to different hygrothermal conditions. It was reported that the FCSM sheet has a high resistance toward the tension-tension fatigue loading [40]. Recognizing this, Lam et al. [41] strengthened deep reinforced concrete (RC) beams using FCSM sheets in an attempt to enhance the shear strength. A substantial increase in the peak sustained load of the RC beams was noted, whereas the shear capacity of the strengthened beams increased by 68% compared to that of the control beam. Bhaskar and Srinivas [42] investigated the performance of FCSM sheets in improving the flexural performance of RC beams. The results indicated the better structural performance of the strengthened beams over that of the control beams.

To date, no detailed study is present to assess the efficacy of FCSM sheets in mitigating the unsatisfactory mechanical characteristics of recycled aggregate concrete (CRA). This study aims to fill this gap by strengthening CRA specimens with FCSM sheets and exploring the enhancement in the mechanical characteristics, mainly the peak compressive stress and the ultimate compressive strain. Given that FCSM sheets offer strength mainly in their axial direction, the possibility of using existing compressive stress-strain analytical models for concrete externally confined with FRP in predicting the mechanical characteristics of CRA is also investigated. For this purpose, this study presented experimental findings of the monotonic compression tests applied to concrete constructed with CRA and externally confined with low-cost FCSM wraps. Three concrete strengths were considered, and eight rectilinear specimens were tested for each concrete strength. For each concrete strength, two, three, and four wraps of FCSM were applied.

## 2. Experimental Program

### 2.1. Test Matrix

Twenty-four specimens were constructed and tested in this study. Specimens were categorized into three groups depending on the concrete strength (see Table 1). The design strength of the concrete in groups 1, 2, and 3 was 15, 20, and 25 MPa, respectively. Specimens in each group were of four types, and two specimens belonged to each particular type to assess the consistency of the results. The first type comprised two control specimens, the second type comprised two specimens strengthened using two wraps of FCSM confinement, and the third specimens were strengthened using three wraps, whereas the fourth type specimens were strengthened using four FCSM wraps. The notation for each specimen recognized its concrete strength, the presence of FCSM sheets, and the number of their wraps. For this, the first part corresponded to 15, 20, or 25 MPa concrete, respectively. The second part was either CON or FCSM corresponding to the control or strengthened specimens, respectively. The last part described the number of FCSM layers. For instance, 20-FCSM-2L represented a specimen constructed with 20 MPa concrete strength and strengthened using two layers of FCSM wraps.

### 2.2. Material Properties

**Figure 1:**
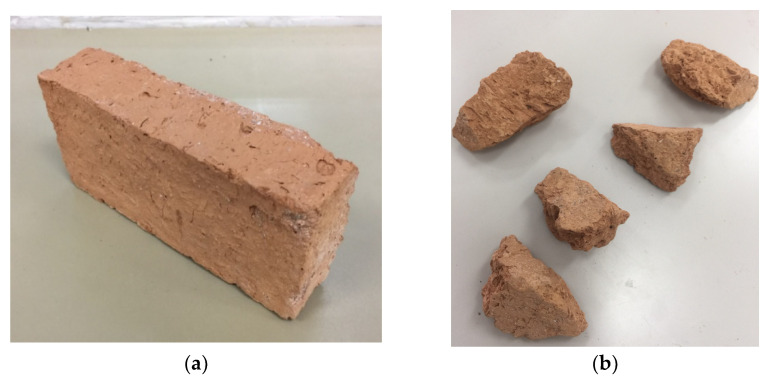
(**a**) Solid clay brick, and (**b**) recycled brick aggregates.

**Figure 2:**
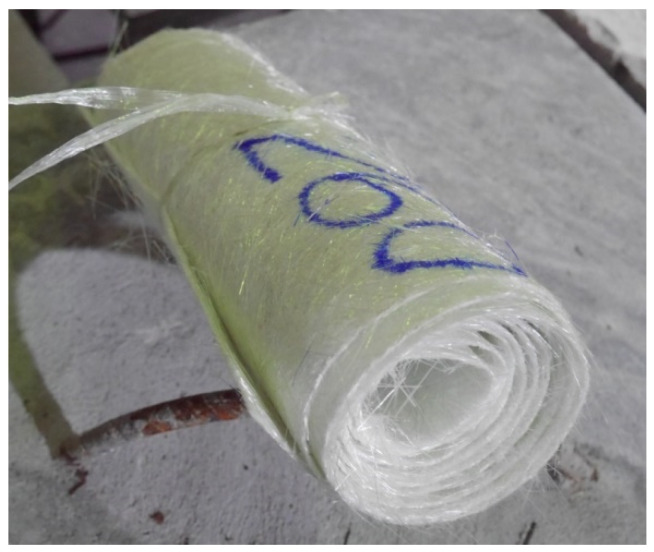
Typical chopped strand mat.

Aggregates were recycled by crushing solid clay bricks (see Figure 1a) using a brick crushing machine, as shown in Figure 1b. Screening of the crushed bricks was performed, resulting in brick aggregates with sizes from 5 mm–20 mm. The recommendations of ASTM C1314-21 and ASTM C140/C140M-22a [43,44] were used to measure the mechanical characteristics of the bricks, such as water absorption, density, and compressive capacity. The density of the bricks was estimated at 120 $\mathrm{kg}/m^{3}$, compressive capacity at 3.14 MPa, and water absorption at 23.27%. Concrete was prepared by substituting 50% of the natural coarse aggregates with recycled brick aggregates. The mix proportions of the concrete for three design strengths are presented in Table 2. In this study, the FCSM sheet was comprised of a non-woven glass fiber mat manufactured by spreading a continuous filament roving of 50 mm in length randomly in combination with a polyester binder (Figure 2). The density of the FCSM sheet was 600 g/m^2^. The thickness of the FCSM sheet was 0.5 mm and the width of the FCSM roll was 1.0 m. The mechanical properties of the FCSM wraps were estimated by following the recommendations of ASTM D3039M-08 [45]. The ultimate tensile strength and modulus of elasticity of the FCSM composite sheet were estimated as180 MPa and 7470 MPa.

### 2.3. Typical Specimen Details, Fabrication, and Strengthening Process

**Figure 3:**
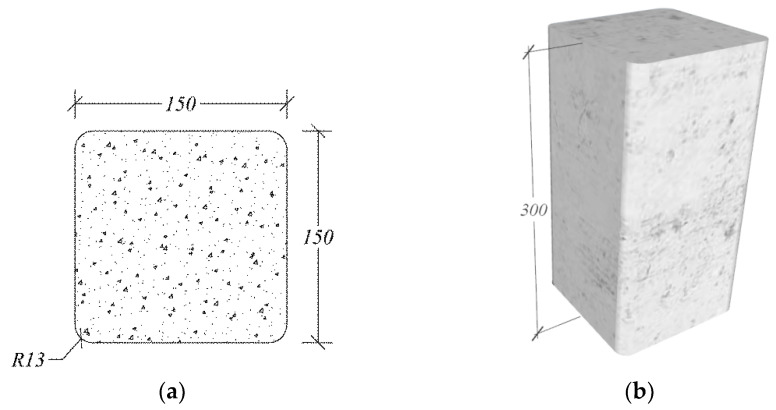
Typical specimen details (**a**) cross-section, and (**b**) 3D view.

In this study, rectilinear concrete specimens of dimensions of 150 mm×150 mm×300 mm were constructed, as shown in Figure 3. The sharp corners were rounded off to a 13 mm radius in accordance with ACI 440.2R-17 [46] to improve the efficiency of the FCSM wraps by reducing the stress concentrations near the sharp corners.

**Figure 4:**
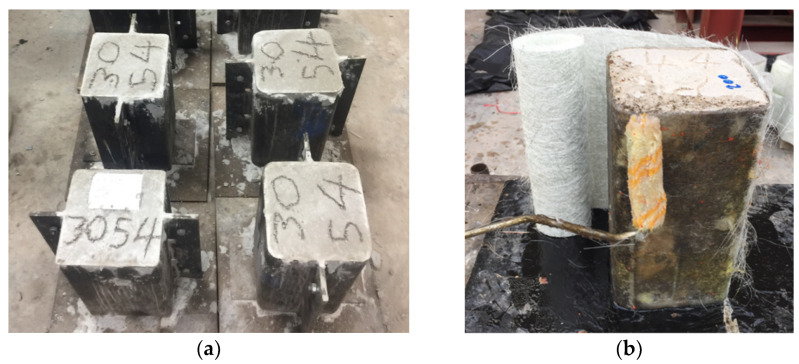

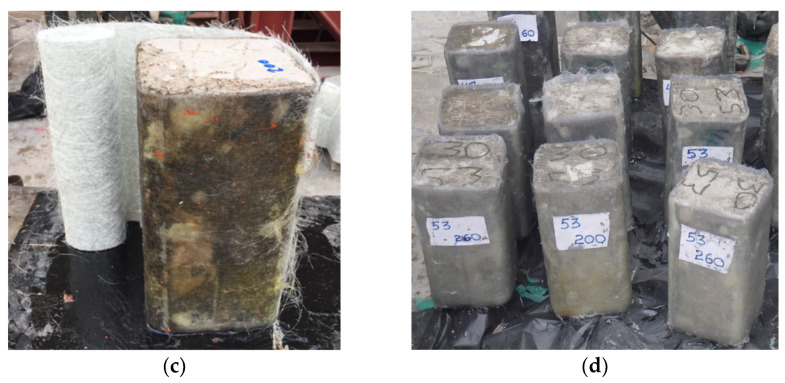
(**a**) Steel molds, (**b**) application of resin using a roller, (**c**) FCSM wrapping in progress, and (**d**) strengthened specimens.

All specimens were constructed in laboratory environments. Steel molds were prepared to cast the specimens, as shown in Figure 4a. Concrete pouring was performed in three equal layers. Each individual concrete layer was compacted using vibration tables to achieve uniform compaction. Steel molds were taken off following one day of casting, whereas the curing of the specimens was maintained for 28 days. Each specimen was strengthened after complete curing of 28 days. Specimens were prepared by thoroughly cleaning their surfaces using cloth, and rough patches were removed before the application of the FCSM wraps. Further, a brush was used to apply epoxy and then a roller was used to remove the entrapped air between the concrete surface (see Figure 4b) and the FCSM composite. For the next thickness, the surface was thoroughly soaked with resin, followed by the application of the FCSM wrap, as shown in Figure 4c. FCSM sheets were tightened during their application to ensure uniform contact with the concrete surface. An analogous process was performed to attach the subsequent FCSM wraps. Typical FCSM strengthened specimens are shown in Figure 4d. The interfacial interactions between the concrete and FCSM as well as the FCSM-FCSM were assumed to be perfectly bonded because the concrete surface and or the FCSM were thoroughly soaked with the resin prior to the next layer of the FCSM sheet.

### 2.4. Test Setup and Instrumentation

**Figure 5:**
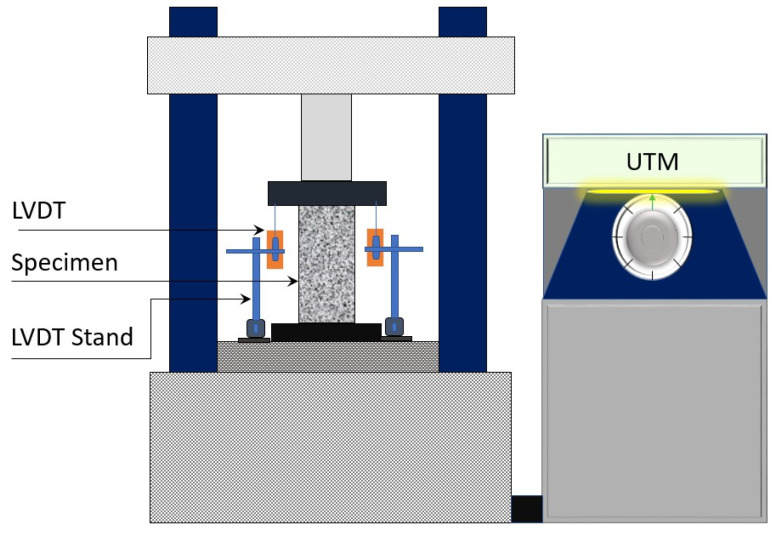
Typical test setup.

A universal testing machine (UTM) with a 1000 kN was utilized to apply a compressive monotonic load. The end surfaces of each specimen were properly cleaned and smoothened prior to the testing. Steel plates were attached above and beneath the specimen to guarantee a uniform load application. A load cell with a 500 kN capacity was utilized to measure the load intensity, whereas a logger was used to record the measured data. Two linear variable displacement transducers (LVDT) were employed to measure the compressive shortening of the specimens (see Figure 5).

## 3. Experimental Results

### 3.1. Failure Modes

**Figure 6:**
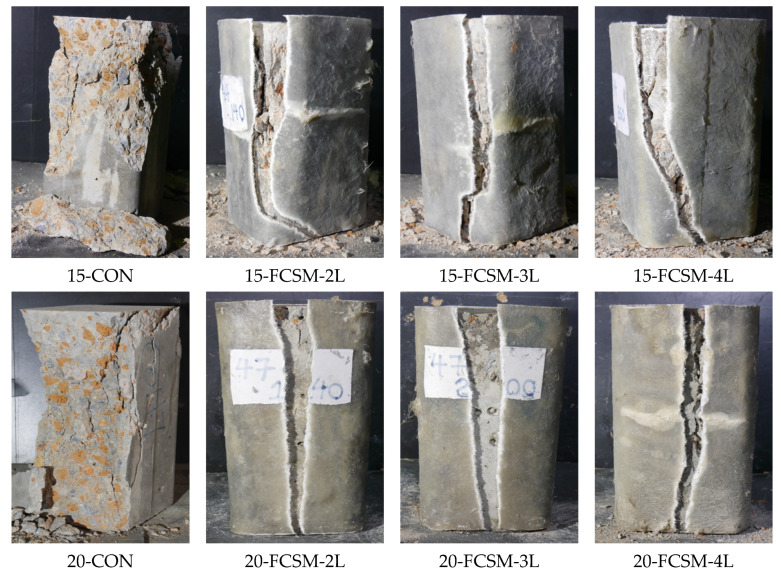

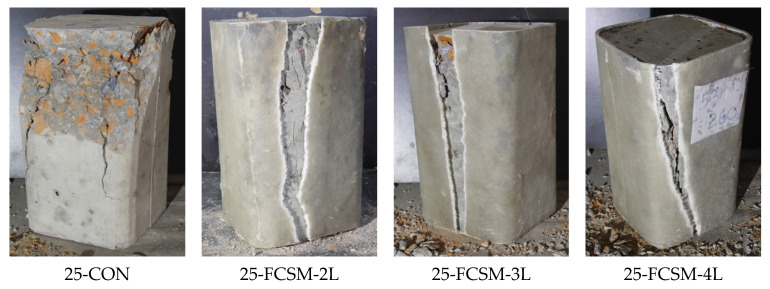
Failure modes.

The failure types of specimens in each group are shown in Figure 6. Specimen 15-CON failed due to the splitting and crushing of the concrete. The crushing was concentrated within its upper half. Specimen 15-FCSM-2L exhibited a delayed and less brittle failure as compared to Specimen 15-CON (Further discussions on this delayed behavior are provided in Section 3.3). The failure of Specimen 15-FCSM-2L accompanied the tearing of the FCSM wraps in the hoop direction, whereas the rupture was mainly concentrated near the corners. This indicates that the 13 mm corner radius was insufficient to mitigate the stress concentrations completely. Specimen 15-FCSM-3L also failed due to the rupture of the FCSM wraps near the corners. However, the concrete crushing was lesser than that of Specimen 15-FCSM-2L, and the failure mode was less brittle as well. Finally, Specimen 15-FCSM-4L exhibited the least brittle failure among the group 1 specimens, and the least concrete crushing was observed. However, the rupture of the FCSM wraps was still concentrated in the corners.

Specimen 20-CON failed in a brittle manner similar to Specimen 15-CON. However, the crushing and splitting of the concrete were detected along its full height. The failure of strengthened specimens in group 2 (i.e., with a 20 MPa designed concrete strength) also accompanied the rupture of the FCSM wraps. However, this rupture was observed in the center of the vertical sides. This suggests that the 13 mm corner radius was sufficient in the higher strength concrete. The ultimate failure modes of the group 3 specimens were similar to those in group 2, as shown in Figure 6.

### 3.2. Peak Stress and Ultimate Strain

The experimental peak compressive stresses and ultimate strains are presented in Table 3. The increase in the peak compressive stress as a result of two, three, and four FCSM wraps in the first group was 61%, 98%, and 140%, respectively. The increase in the ultimate strain was 188%, 270%, and 320%, respectively for the same specimens. For the second group, two, three, and four FCSM wraps increased the peak compressive stress by 53%, 74%, and 102%, respectively, whereas the improvement in the ultimate strain was 163%, 255%, and 308%, respectively. Similarly, the increase in the peak compressive stress of the third group specimens as a result of two, three, and four FCSM wraps was 46%, 65%, and 83%, respectively, whereas the ultimate strain improved by 135%, 235%, and 294%, respectively. Both the peak compressive stress and the ultimate strain were substantially increased due to FCSM confinement regardless of the strength of concrete and the number of FCSM wraps. The issues associated with the effect of concrete strength and the quantity of FCSM wraps on the efficacy of the FCSM wraps are discussed in the subsequent sections.

### 3.3. Compressive Stress-Strain Curves

**Figure 7:**
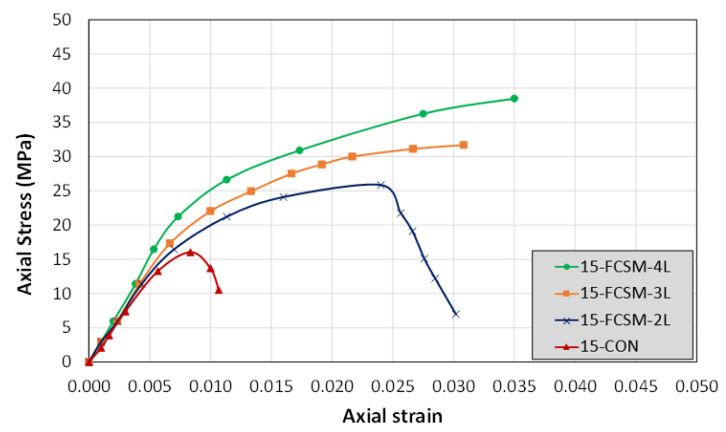
Compressive stress-strain curves of group 1 specimens.

Continuous recording of the compressive load and axial shortening was conducted using a data logger. The recorded compressive load was converted to the compressive stress using the cross-sectional area of the specimens, whereas the compressive shortening to the strain us was converted ing the height of the specimens. The measured compressive stress and strain curves of the group 1 specimens are illustrated in Figure 7. The control Specimen 15-CON exhibited typical stress versus strain response of unconfined concrete. A steep ascending branch was observed till a peak value of about 16.0 MPa, followed by an abrupt drop due to the brittle failure. The specimen 15-FCSM-2L was able to sustain high ultimate strains to a value of 0.024. At this point, the sudden rupture of the FCSM wraps led to a drop in its compressive load capacity. The specimens strengthened with 3, and 4 FCSM wraps exhibited a bilinear stress-strain response exhibiting a high ductility till the ultimate strains of 0.0308 and 0.035, respectively.

**Figure 8:**
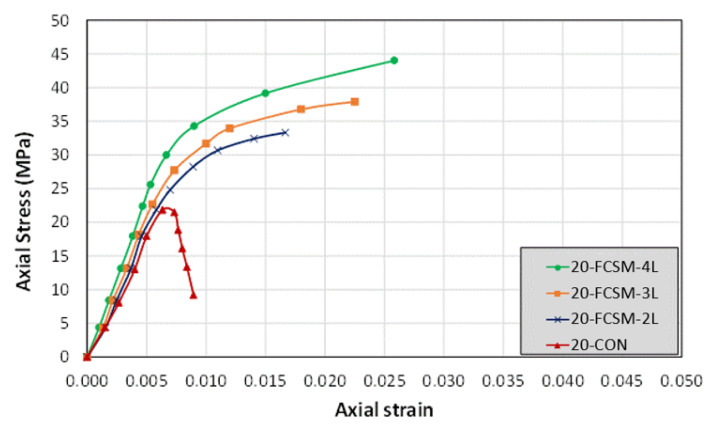
Compressive stress-strain curves of group 2 specimens.

The stress versus strain graphs of the group 2 specimens are shown in Figure 8. The control Specimen 20-CON failed in a brittle manner dropping its load capacity abruptly, and did not exhibit any ductility. All the strengthened specimens in group 2 demonstrated a bilinear response. The peak compressive stress and the ultimate strain sustained improved with the number of FCSM wraps. The ductility of the CRA was observed to increase with the number of FCSM wraps as well. Unlike Specimen 15-FCSM-2L, Specimen 20-FCSM-2L did not drop its capacity, which can be attributed to the higher unconfined concrete strength in group 2.

**Figure 9:**
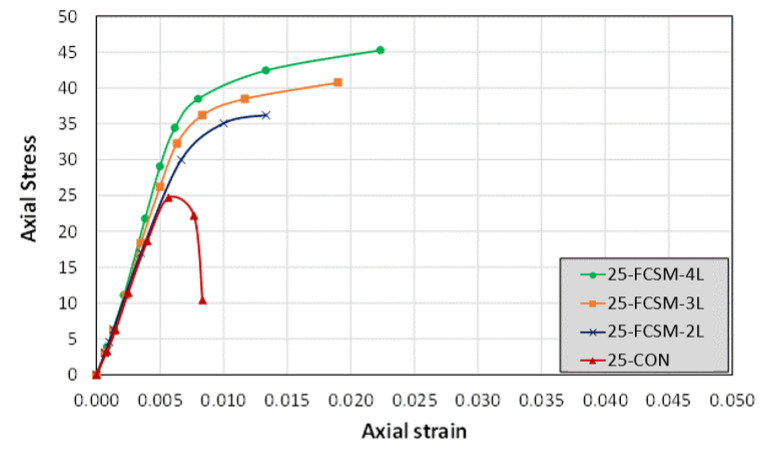
Compressive stress-strain curves of group 3 specimens.

Finally, the stress versus strain graphs of the group 3 specimens are presented in Figure 9. The stress-strain response was similar to those of the specimens in group 2. The strengthened specimens depicted a bilinear response, whereas the control Specimen 25-CON failed abruptly. It is clear that the FCSM sheets provided sufficient compressive ductility to the CRA. For the 25 MPa concrete strength, two FCSM wraps enhanced the peak load up to a certain strain level only. Apart from Specimen 15-FCSM-2L, all the confined specimens exhibited bilinear stress versus strain behavior.

### 3.4. Effect of the Number of FCSM Wraps and Concrete Strength

**Figure 10:**
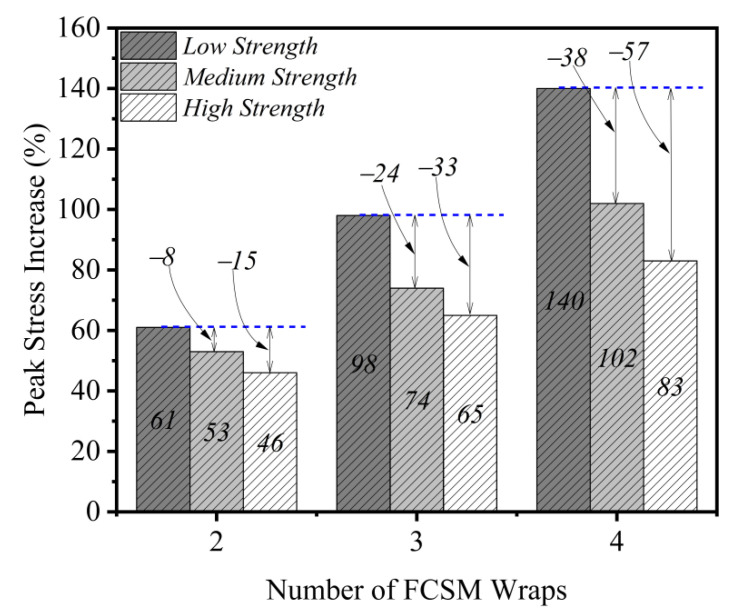
Effect of FCSM wraps and concrete strength on the increase in peak compressive stress.

The effect of the number of FCSM wraps and concrete strength on the increase in the peak compressive stress is shown in Figure 10. It can be seen that by increasing the number of FCSM wraps, a clear improvement was detected in the peak compressive stress. For the low-strength specimens, this increase was 61%, 98%, and 140% for 2, 3, and 4 FCSM wraps, respectively. The corresponding increase in the peak stress for the group 2 specimens was found to be lower than that for group 1 specimens. A further reduction in the increase in the peak compressive stress was observed for the group 3 specimens. This is indicated in Figure 10 as for the two wraps of the FCSM, the increase in the peak stress for the medium and high-strength concrete was 8% and 15% lower than that of the low concrete strength specimens. This difference increased as the number of FCSM wraps increased to three, where the medium and high-strength specimens experienced an increase in the peak stress that was lower than that of the low-strength specimen by 24% and 33%, respectively. This difference further increased as the number of FCSM wraps increased to four, where the medium and high-strength specimens experienced a 38% and 57% lower increase in the peak stress as compared to the low-strength specimen.

**Figure 11:**
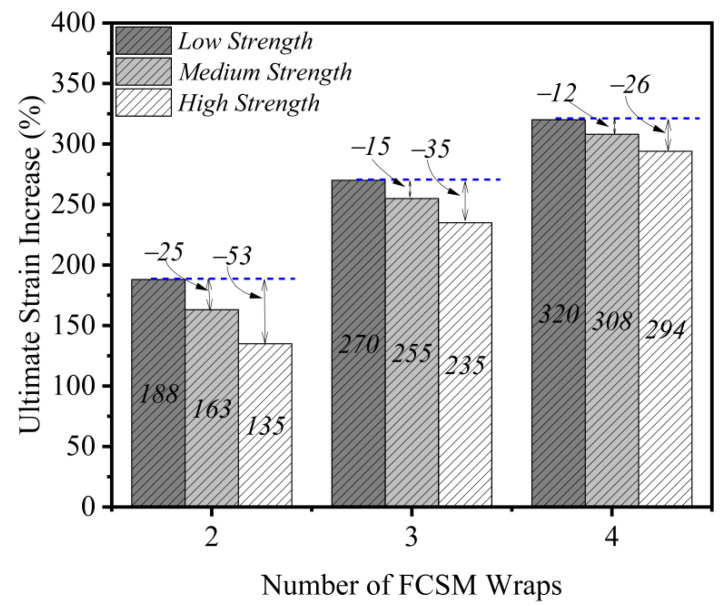
Effect of FCSM wraps and concrete strength on the increase in ultimate strain.

The impact of the strength of concrete and the number of FCSM wraps on the increase in the ultimate strain is shown in Figure 11. The maximum gain in the ultimate strain was detected for the low concrete strength specimens, irrespective of the number of FCSM wraps. This was followed by the medium and high concrete strength specimens, respectively. This observation is analogous to the one made for the peak stress improvement in Figure 10. However, the difference in the gain in the peak stress increased as the number of FCSM wraps increased (see Figure 10), whereas this difference decreased for the case of the ultimate strain (see Figure 11). For instance, the difference in the gain of the ultimate strain between the low and medium concrete strength specimens for two FCSM wraps was 25%, whereas this difference was reduced to 15% and 12% for the case of three and four FCSM wraps. In general, both the peak compressive stress and ultimate strain improved as the number of FCSM wraps increased, whereas this improvement was reduced as the unconfined concrete strength increased.

## 4. Analytical Investigations

### 4.1. Existing Analytical Models

**Figure 12:**
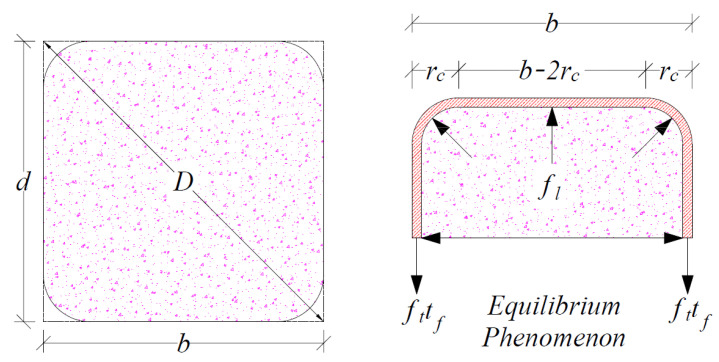
Equilibrium between the outward bursting pressures and axial forces in FCSM wraps.

In this section, existing analytical models were evaluated in approximating the peak compressive stress and ultimate strain. To the authors’ knowledge, no analytical models for FCSM confined concrete are available in the literature. However, several researchers have proposed confinement models for synthetic and natural fiber-reinforced polymer (FRP) confined concrete [25,47,48,49,50,51,52,53,54,55]. Since FCSM and FRP wraps exert confinement pressures through their in-plane stiffness mainly, it is assumed that existing analytical models can be applied to FCSM confined concrete. In the existing analytical studies, the general form of Equation (1) [56] is used to relate the peak compressive stress of strengthened concrete $f_{cc}$ to the lateral pressure $f_{l}$ applied by external wraps.

(1) $$\frac{f_{cc}}{f_{co}}=1+k_{1}\frac{f_{l}}{f_{co}}$$

where $f_{co}$ is the unconfined compressive strength and the constant $k_{1}$ is proposed from the regression. From the equilibrium between the outward bursting pressure $f_{l}$ under the compressive loads and the resulting forces $f_{t}\times t_{f}$ in the FCSM wraps shown in Figure 12, an expression for $f_{l}$ can be derived in the form of Equation (2) [47,48,50,51,52,53,54,55]. This is the general form of the equation for confining pressure. This equation has been extensively used in previous studies [47,48,50,51,52,53,54,55].

(2) $$f_{l}=\frac{2n_{f}f_{t}t_{f}}{D}\rho$$

where D is the length of the diagonal of the rectilinear section given in Equation (3) [46], $f_{t}$ is the ultimate tensile capacity of the FCSM sheet, $t_{f}$ is the thickness of the FCSM sheet, $n_{f}$ is the number of wraps, and ρ can be determined using Equation (4) [57].

(3) $$D=\sqrt{b^{2}+d^{2}}$$

(4) $$\rho =1-\frac{{\left(b-2r_{c}\right)}^{2}+{\left(d-2r_{c}\right)}^{2}}{3A}$$

where b and d are the cross-sectional sizes of the section defined in Figure 12, $r_{c}$ is the corner radius, and A is the gross area defined in Equation (5) considering the corner radii.

(5) $$A=bd-\left(4-\pi \right)r_{c}^{2}$$

Several existing peak stress and ultimate strain models are described in Table 4.

The accuracy of the analytical models in Table 4 is evaluated by the mean value (referred to as the AVG) of the ratios of the predicted to the experimental peak compressive stresses and the corresponding standard deviations STDs. The average and standard deviations for the peak compressive stresses are presented in Table 5. The considered models underestimated the peak compressive stresses of the CRA confined with the FCSM. This is indicated by their AVG values of less than 1.0 in Table 5. However, it was observed that the AVG values increased for a particular model as the unconfined compressive strength increased. For instance, the model of Shehata et al. [25] resulted in AVG values of 0.56, 0.62, and 0.65 for groups 1, 2, and 3, respectively. The inconsistency of the existing models to approximate the peak compressive stress of the FCSM strengthened CRA with different unconfined concrete strengths suggests that there is a need for the analytical model that could consider the impact of the unconfined concrete strength without comprising the consistency of an accurate prediction of the peak compressive stresses.

The existing models were evaluated to predict the ultimate strain of the CRA confined with FCSM wraps. For the low-strength specimens in group 1, the models of ACI-440.2 R-17 [46] and Lam and Teng [51] produced AVG values of 0.81 and 1.02, respectively. The corresponding standard deviations were 0.107 and 0.030. For group 2, the best prediction was provided by Ilki and Kumbasar [58], with an AVG value of 1.00 and a standard deviation of 0.049. Finally, the ultimate strain of group 3 strengthened specimens was best predicted by the models of ACI-440.2 R-17 [46], Lam and Teng [51], and Ilki and Kumbasar [58]. However, they produced standard deviations of 0.071, 0.123, and 0.097, respectively. This suggests that none of the considered models predicted the ultimate strain of strengthened specimens of all groups consistently.

### 4.2. Proposed Models

**Figure 13:**
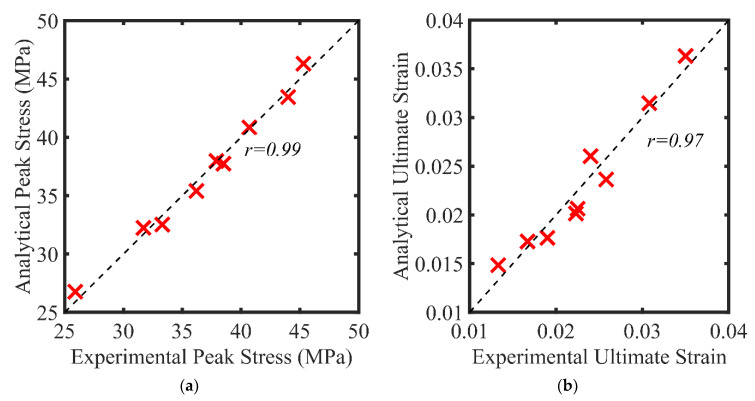
Comparison of experimental versus predicted (**a**) peak compressive stress and (**b**) ultimate strain.

The experimental results were utilized to propose expressions to estimate the peak compressive stress and ultimate strain of the CRA strengthened with FCSM wraps. A nonlinear regression was performed to propose Equations (6) and (7) for the peak stress and ultimate strain of the CRA strengthened with FCSM wraps.

(6) $$\frac{f_{cc}}{f_{co}}=1+8.56{\left(\frac{f_{l}}{f_{co}}\right)}^{1.012}$$

(7) $$\frac{\epsilon_{u}}{\epsilon_{co}}=1+11.15{\left(\frac{f_{l}}{f_{co}}\right)}^{0.67}$$

where $f_{co}$ is the compressive strength of the unconfined concrete, $\epsilon_{co}$ is the ultimate strain of the unconfined concrete, and $f_{l}$ is the lateral pressure exerted by the FCSM wraps and computed from Equation (2). The accuracy of the proposed equations is illustrated in Figure 13a and Figure 13b for the peak compressive stress and ultimate stress, respectively. Pearson’s coefficient was utilized to measure the accuracy of the proposed equations and defined using Equation (8).

(8) $$r=\frac{\sum \left(x_{i}-\bar{x}\right)\left(y_{i}-\bar{y}\right)}{\sqrt{\sum {\left(x_{i}-\bar{x}\right)}^{2}{\left(y_{i}-\bar{y}\right)}^{2}}}\;\;\;$$

where $x_{i}$ is the ith observed value and $y_{i}$ is the ith predicted value, $\bar{x}$ is the sample mean of the observed values, and $\bar{y}$ is the sample mean of the predicted values. An r of 0.99 and 0.97 was obtained for Equations (6) and (7), respectively, indicating that high accuracy in predicting the experimental results was obtained. Further, the mean values of the ratios of the predicted to the experimental values and corresponding standard deviations are presented in Table 5 and Table 6. It can be seen in Table 5 that the AVG values of 1.01, 0.99, and 1.00 were obtained for groups 1, 2, and 3 specimens, respectively, whereas the corresponding standard deviations were 0.027, 0.013, and 0.022, respectively. This suggests that the proposed Equation (6) accurately predicted the peak compressive stress of the CRA strengthened with FCSM wraps. Similarly, the AVG values and standard deviations for Equation (7) are presented in Table 6. AVG values of 1.03, 0.97, and 0.99 were produced by Equation (7) for the group 1, 2, and 3 specimens, respectively, whereas the corresponding standard deviations were 0.032, 0.058, and 0.090, respectively.

## 5. Conclusions

1. This study presented experimental findings of the monotonic compression tests applied to concrete constructed with recycled brick aggregates (CRAs) and externally confined with low-cost FCSM wraps. Three concrete strengths were considered, and eight rectilinear specimens were tested for each concrete strength. For each concrete strength, two, three, and four wraps of FCSM were applied. The subsequent important inferences can be made:
2. The peak compressive stress of the specimens was increased by 61%, 98%, and 140% as compared to the reference specimen for the 2, 3, and 4 wraps of the FCSM applied to the low strength (i.e., a 15 MPa design strength) CRA specimens. For the medium strength CRA (i.e., a 20 MPA design strength), an up to 102% improvement in the peak stress was observed, whereas the peak stress was improved up to 83% for the high strength CRA (i.e., a 25 MPa design strength). The peak stress was found to increase as the number of FCSM wraps increased.
3. The FCSM wraps were efficient in enhancing the compressive ductility of the CRA. For the low, medium, and high strength CRA, the ultimate strain improved up to 320%, 308%, and 294%, respectively, as compared to the respective control specimens.
4. In particular, 3 and 4 wraps of the FCSM resulted in a bilinear stress-strain behavior irrespective of the concrete strength.
5. The improvement in the peak stress and ultimate strain as a result of the FCSM wrap confinement varied in inverse relation to the unconfined concrete strength, irrespective of the number of FCSM wraps.
6. Various existing analytical models of confined concrete were assessed to predict the peak compressive stress and ultimate strain of the CRA strengthened with the FCSM wraps. None of the existing models were found to estimate the peak stress and ultimate strain for all the groups consistently. Therefore, equations for the peak stress and ultimate strain were formulated from a nonlinear regression analysis. The accuracy of the proposed equations was assessed using Pearson’s coefficient r. An r value of 0.99 and 0.97 was observed for the equation of the peak stress and ultimate strain, respectively, indicating that a good agreement existed between the experimental and predicted values.

## Figures and Tables

**Table 1:** Test specimens.

ID	15 MPa	20 MPa	25 MPa
15-CON	15	None	2
15-FCSM-2L	15	2	2
15-FCSM-3L	15	3	2
15-FCSM-4L	15	4	2
20-CON	20	None	2
20-FCSM-2L	20	2	2
20-FCSM-3L	20	3	2
20-FCSM-4L	20	4	2
25-CON	25	None	2
25-FCSM-2L	25	2	2
25-FCSM-3L	25	3	2
25-FCSM-4L	25	4	2

**Table 2:** Concrete mix constituents.

$\mathrm{Constituents}\;\left(kg/m^{3}\right)$	15 (MPa)	20 (MPa)	25 (MPa)
Cement	261	438	627
Sand	783	788	806
Natural stone aggregates	522	438	358
Brick aggregates	522	438	358
Water	313	298	251

**Table 3:** Summary of peak compressive stress and ultimate strain.

ID	Peak Stress (MPa)	Increase in Peak Stress (%)	Ultimate Strain	Increase in Ultimate Strain (%)
15-CON	16.0	-	0.0083	-
15-FCSM-2L	25.9	61	0.0240	188
15-FCSM-3L	31.7	98	0.0308	270
15-FCSM-4L	38.5	140	0.0350	320
20-CON	21.8	-	0.0063	-
20-FCSM-2L	33.3	53	0.0167	163
20-FCSM-3L	37.9	74	0.0225	255
20-FCSM-4L	44.0	102	0.0258	308
25-CON	24.7	-	0.0057	-
25-FCSM-2L	36.2	46	0.0133	135
25-FCSM-3L	40.7	65	0.0190	235
25-FCSM-4L	45.3	83	0.0223	294

**Table 4:** Model expressions of existing peak compressive stress and ultimate strain models for confined concrete.

Model	$\mathrm{Peak}\;\mathrm{Compressive}\;\mathrm{Stress}\;f_{cc}$	$\mathrm{Ultimate}\;\mathrm{Strain}\;\epsilon_{u}$
Shehata et al. [25]	$\frac{f_{cc}}{f_{co}}=1+0.85\left(\frac{f_{l}}{f_{co}}\right)$	$\frac{\epsilon_{u}}{\epsilon_{co}}=1+13.5\left(\frac{f_{l}}{f_{co}}\right)$
ACI-440.2 R-17 [46]	$f_{cc}=f_{co}^{’}+\left(0.95\right)\left(3.0\right)\left(\frac{A_{e}}{A_{c}}\right){\left(\frac{b}{d}\right)}^{2}f_{l}$	$\epsilon_{u}=\epsilon_{co}\left(1.50\;+12\left(\frac{A_{e}}{A_{c}}\right){\left(\frac{d}{b}\right)}^{0.5}\left(\frac{f_{l}}{f_{co}^{’}}\right){\left(\frac{\epsilon_{fe}}{\epsilon_{co}}\right)}^{0.45}\right)$
Kumutha et al. [48]	$\frac{f_{cc}}{f_{co}}=1+0.93\left(\frac{f_{l}}{f_{co}}\right)$	-
Al-Salloum [49]	$\frac{f_{cc}}{f_{co}}=1+3.14\left(\frac{b}{D}\right)\left(\frac{f_{l}}{f_{co}}\right)$	-
Mirmiran et al. [50]	$\frac{f_{cc}}{f_{co}}=1+6.0\left(\frac{2r_{c}}{D}\right)\left(\frac{f_{l}}{f_{co}}\right)$	-
Lam and Teng [51]	$\frac{f_{cc}}{f_{co}}=1+3.30\left(\frac{f_{l}}{f_{co}}\right)$	$\frac{\epsilon_{u}}{\epsilon_{co}}=1.75+12.0\left(\frac{f_{l}}{f_{co}}\right){\left(\frac{\epsilon_{fe}}{\epsilon_{co}}\right)}^{0.45}$
Pimanmas et al. [52]	$\frac{f_{cc}}{f_{co}}=1+2.50\left(\frac{f_{l}}{f_{co}}\right)$	$\frac{\epsilon_{u}}{\epsilon_{co}}=2+7.0\left(\frac{f_{l}}{f_{co}}\right)$
Ilki and Kumbasar [58]	$\frac{f_{cc}}{f_{co}}=1+2.227\left(\frac{f_{l}}{f_{co}}\right)$	$\frac{\epsilon_{u}}{\epsilon_{co}}=1+15.0{\left(\frac{f_{l}}{f_{co}}\right)}^{0.75}$

Note: $\epsilon_{co}=$ ultimate strain of unconfined concrete, and $\epsilon_{fe}=$ effective strain of external wrap (taken as 0.6 times the ultimate strain), *A_c_* = cross-sectional area of concrete in compression member and *A_e_* = cross-sectional area of effectively confined concrete section.

**Table 5:** Assessment of analytical models to predict peak compressive stress of FCSM confined CRA.

Model	Group 1		Group 2		Group 3	
	AVG	STD	AVG	STD	AVG	STD
Shehata et al. [25]	0.56	0.093	0.62	0.071	0.65	0.060
ACI-440.2 R-17 [46]	0.50	0.104	0.57	0.083	0.60	0.073
Kumutha et al. [48]	0.57	0.093	0.62	0.070	0.65	0.059
Al-Salloum [49]	0.70	0.074	0.73	0.048	0.76	0.035
Mirmiran et al. [50]	0.57	0.092	0.63	0.069	0.66	0.057
Lam and Teng [51]	0.71	0.073	0.74	0.047	0.77	0.033
Pimanmas et al. [52]	0.66	0.080	0.70	0.055	0.73	0.042
Ilki and Kumbasar [58]	0.65	0.082	0.69	0.057	0.72	0.045
Proposed Equation (6)	1.02	0.023	0.99	0.011	1.00	0.020

**Table 6:** Assessment of analytical models to predict ultimate strain of FCSM confined CRA.

Model	Group 1		Group 2		Group 3	
	AVG	STD	AVG	STD	AVG	STD
Shehata et al. [25]	0.73	0.023	0.65	0.032	0.66	0.066
ACI-440.2 R-17 [46]	0.81	0.107	0.78	0.081	0.70	0.071
Lam and Teng [51]	1.02	0.030	0.98	0.070	1.03	0.123
Pimanmas et al. [51]	0.80	0.077	0.78	0.116	0.83	0.166
Ilki and Kumbasar [58]	1.13	0.023	1.00	0.049	1.03	0.097
Proposed Equation (7)	1.03	0.033	0.96	0.061	0.99	0.091
